# Mental health services conceptualised as complex adaptive systems: what can be learned?

**DOI:** 10.1186/s13033-017-0150-6

**Published:** 2017-06-29

**Authors:** Louise A. Ellis, Kate Churruca, Jeffrey Braithwaite

**Affiliations:** Centre for Healthcare Resilience and Implementation Science, Australian Institute of Health Innovation, Macquarie University, Level 6, 75 Talavera Rd, North Ryde, NSW 2109 Australia

**Keywords:** Mental health services, Complex adaptive systems, Australia

## Abstract

Despite many attempts at promoting systems integration, seamless care, and partnerships among service providers and users, mental health services internationally continue to be fragmented and piecemeal. We exploit recent ideas from complexity science to conceptualise mental health services as complex adaptive systems (CASs). The core features of CASs are described and Australia’s *headspace* initiative is used as an example of the kinds of problems currently being faced. We argue that adopting a CAS lens can transform services, creating more connected care for service users with mental health conditions.

## Introduction

Despite many attempts at promoting systems integration and partnerships among service providers and users [1], mental health services in most countries continue to be fragmented, with disjointed professional groups working within their own mental models and inside their own silos [2]. Cross-disciplinary, inter-professional and inter-organizational working is often lacking [3]. Internationally, governments have recognised the need to ‘think differently’ about mental health policy and service delivery [4], and there is growing appreciation for systems thinking, particularly from the broader health care sector [5]. In this paper, we exploit recent ideas from complexity science to conceptualise mental health services as *complex adaptive systems* (CASs) [6]. First, we look at what fragmented mental health care looks like, and then apply CAS ideas to the *headspace* program, a flagship initiative in Australia. This paper aims to contribute to growing discourse regarding the value of adopting a CAS perspective for increasing our understanding of the problems currently being faced in the mental health sector and as a future guide to the types of efforts needed to create better connected care.

### Traditional arrangements for mental health care

In traditional mental health care in Australia, paralleling many systems of care elsewhere, a person typically visits his or her primary-care provider (e.g., general practitioner or family doctor), is given a referral, and often waits for weeks to be assessed by a clinician before treatment can commence. The service-seeker is likely to move between providers for different services, hoping to secure the care he or she needs. Eventually, he or she might have reached the right type of professional or mix of care required, commensurate with the predisposing condition. Meanwhile, the person needs to retell their story, with those asking for the information themselves coming from different perspectives or making differing assumptions about the information being provided. Navigating the system is a formidable challenge for the service user, and communication and interaction between professionals typically limited, often posing unnecessary risks to the system and the recipient. People with mental health problems get lost in the system, leaving themselves and their families vulnerable to significant health and social risks [2].

Modern health care is complex, and mental health care particularly so [4]. In Australia, as with other systems of care elsewhere, political, social, historical and other factors have led to particularly complex divisions of service provision by an array of health care providers, situated in both acute and non-acute settings. As Hannigan and Coffey [4] highlight, mental health is a “particularly untamed field”, characterised by “too little understanding of the disease, lack of suitable and/or available treatments, poorly trained and/or too few workers, too few and/or the wrong types of teams or facilities…mental health laws which are either too liberal or too coercive” which all add bureaucratic, regulatory and structural complexity (p. 223).

**Fig. 1:**
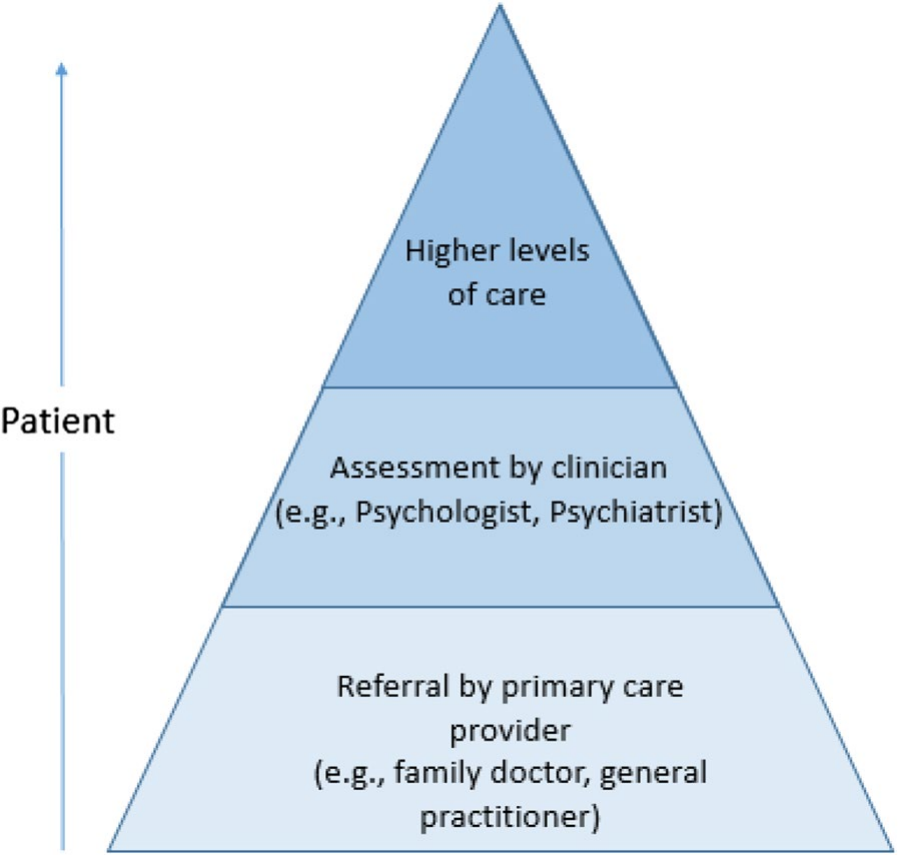
Traditional linear model of mental health care delivery

**Fig. 2:**
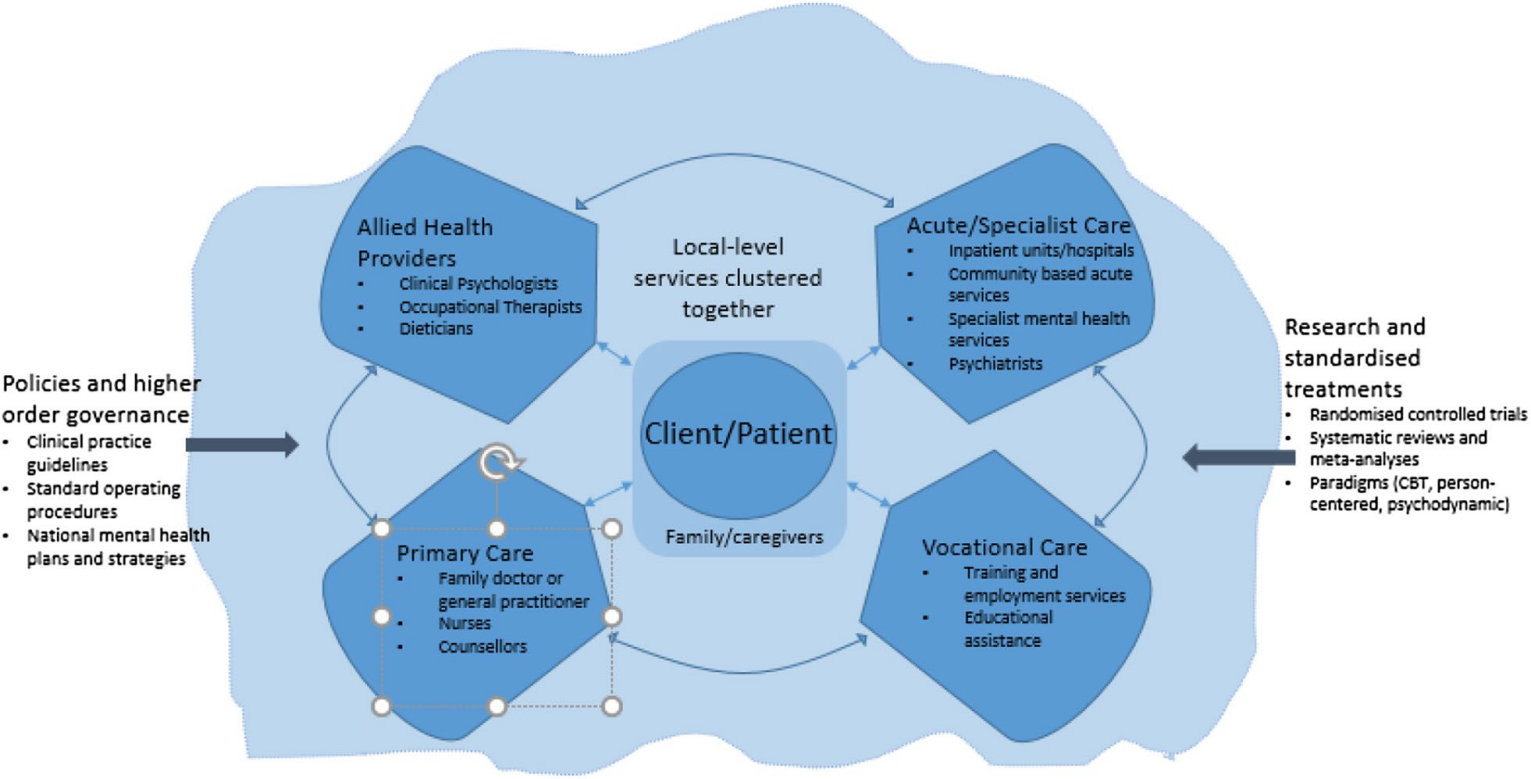
Providers and services grouped together in new arrangements

More recently, the focus has shifted to the interconnections between mental health, physical health and social wellbeing, resulting in attempts to break down the barriers between services [2]. This stance has various names (e.g., ‘collaborative’, ‘multidisciplinary’, ‘coordinated’ and ‘integrated’ care) [7, 8]. Each descriptor has shades and nuances, but we simply make the point that such approaches have led to greater emphasis on the grouping together of care providers including: primary care and general practitioners; providers of housing, employment services, education and training, and related support services; as well as families and carers [9]. In one sense the system is being re-conceptualised, shifting away from viewing it in a segmented, linear way (Fig. 1), to seeing it as a complex system with non-linear pathways and synergistic components (Fig. 2).

### A complex adaptive systems perspective

The move to clustering different types of care providers has led to a greater emphasis in mental health policy on service coordination and service partnerships. However, governments must recognise that making such changes has unpredictable effects and that things often don’t change as planned [10]. It is in this context that we promote the adoption of a CAS perspective to guide thinking in the mental health sector. Australia’s mental health system has arguably become more complex than ever. It was always complicated in the everyday sense of being multi-staged, but now the extended system is being harnessed to provide more pluralist, connected care, with changing relationships between professionals and with service users, as well as the rise of new technologies, so more than ever it is being revealed as a complex adaptive system (CAS) [11].

CASs have been researched in fields as diverse as mathematics, sociology, marketing, science and psychology. By definition, CASs are more than just complicated; they have many defining properties, such as intricate, open, interactive sub-systems with fuzzy boundaries. They are comprised of numerous, diverse, interacting agents [12]. The patterns of relationships and adaptations of the agents contribute to unpredictable, emergent behaviours and events.

More recently, CAS thinking has been applied to health care [13, 14], and is increasingly being used as a conceptual lens in the published literature through which we can identify and solve problems. As an example, Edgren and Barnard examined how a CAS approach can be used to promote the integration of health and social care to the benefit of service users [10]. However, the literature has not, to any substantial extent, examined how the specific characteristics of CASs relate to mental health systems.

First, CASs are made up of active *agents* that are both actors and information processors—in mental health this includes individuals (e.g., doctors, mental health providers, clients), services and organizations (e.g., hospitals, community mental health services, mental health branches of health departments, NGOs, e-mental health services). Agents interact with one another; hence, *interconnections* is the second CAS characteristic. Connections occur at multiple levels (e.g., amongst policymakers, health professionals and mental health service providers, as well as across groups, clustering together to form cultures, networks and hierarchies). Agents influence each other, directly or indirectly through these connections, and their behaviours *coevolve*.

Third, agents in a CAS self-organize around a core driver (in technical terms, an *attractor*), are sensitive to contexts, and their behaviours are *non*-*linear*. In other words, the relationships of interacting stakeholders are dynamic across time, and not particularly predictable. Instead individual agents largely self-organize, conducting their work through their training and their own internalised principles, rather than adhering strictly to top-down policies that ostensibly “manage” their role. Fourth, behaviours in the system *emerge*, meaning agents’ localized interactions form into complex social patterns, such as intricate local rules, structures and cultural features.

An example, demonstrating that the provision of holistic and coordinated services is not easy to achieve, is provided. It shows that adopting a CAS lens can enrich our understanding of the problems being faced and the kinds of efforts needed to create better-connected mental health care.

### The Australian example: headspace

One of Australia’s key responses to providing holistic services for young people aged 12–25 years is the *headspace* program [15]. Unlike traditional mental health service options, *headspace* is intended to provide integrated services across four domains: mental health; primary care; drug and alcohol use; and social, vocational and educational participation. Care is intended to be youth-friendly and highly accessible, providing a multidisciplinary ‘one-stop shop’, closely linked to locally available specialist services, schools and other community-based organizations. Professionals are co-located in an attempt to enhance collaboration, break down professional silos and reduce care fragmentation. Starting with ten centres initially, the network of shop-front clinics has expanded to around 100, covering most of the country, and supported by a National Office.

Two independent evaluations of *headspace* have been published [16, 17]. Progress has been criticized as “disappointing” [18] with only minimal improvement in young people’s mental health being reported [16], despite this integrated care model. So, notwithstanding it being intended to be a major reconfiguration, why isn’t *headspace* doing better? One problem is natural service variation: some centres have been more successful in providing multidisciplinary services than others. Muir et al. (2009) argued that simply co-locating different services has not automatically resulted in well-coordinated care, with one *headspace* provider indicating: “the model is designed to be holistic and while it brings together the practitioners [from different backgrounds], the communication still tends to be in silos… [providers] continue to work individually” [17]. From a CAS perspective, this demonstrates the unpredictable effects of such top-down interventions, and reinforces that it is simplistic merely to co-locate services and professionals with differing approaches, cultures, internalized principles, and expect integrated care simply because management demands it.

### Applying CAS thinking to mental health needs

That being so, can adopting a CAS perspective provide fresh pointers to the kinds of efforts that are needed to improve the state of such services? From a CAS vantage point, an *attractor* is a force that draws the system toward a goal. Applying this idea, a ‘shared vision’ of the mental health system is needed. This should be the service user’s vision not the provider’s, as the user is the only part of the system that experiences all of it. The system needs to be re-oriented towards people retaining or regaining their experience of good mental, physical and social health. However, this goal differs from current policy and practice which largely still focuses on signs and symptoms for diagnosis and implementation of standard treatments [19]. In taking a CAS approach local agents would respond adaptively, not prescriptively. Individual care needs would be personalised, being informed (but not dominated) by research and standard treatment protocols; thus, allowing the emergence of tailored solutions for individuals and communities [19]. To achieve a vision aligned to CAS approaches, service providers would *co*-*work* rather than merely co-locate, adopting a new rule that all professionals see themselves as part of a team in partnership with service users to improve health [10]. This would be predicated on *coevolution*; developing a shared ethos, understanding others’ language and jargon, and coordinating the actions of the team [20].

A CAS-inspired approach would focus on adapting more emergent ways of working instead of prescriptive, excessively planned approaches to change [10]. Indeed, the *headspace* program has been criticised for becoming the “McDonalds version of health care” with the National Office being accused of “being obsessed with brand and marketing” and leaving centres with “no capacity to respond to the unique needs of their local area” [21]. Interviews with *headspace* staff suggest that the model has been developed as “one size fits all … [with limited] … capacity to be flexible around different needs” [16]. Further, the program has been condemned for delivering information in a “paternalistic, non-collaborative way”, and for viewing all centres as “homogenous” [8]. Rather than such top-down mandating, CAS theory recognizes that creative progress can emerge from only a few, flexible rules [14].

Adopting a CAS perspective recognizes the ability of agents to *self*-*organize* around clients’ needs. Organizational arrangements are not decreed into existence but emerge through processes of local negotiation, without excessive centralised control. Complexity science suggests CASs cannot be forced, and top-down attempts to control the system are often counterproductive. Instead CASs require direction without directives.

Thus, to achieve coordinated care, an environment must be created that fosters connectivity among mental health service providers, providing them with sufficient autonomy to respond adaptively to community needs. Through dynamic interactions over time, creative solutions will emerge based on collective insight, distributed control and learning. This ‘hands-off’ approach is in stark contrast to most modern bureaucracies, including that of the *headspace* National Office, which has been criticised for imposing change via direct authority rather than supporting bottom-up collaboration [22]. The recent move by the Australian government to devolve control of *headspace* services to localised primary health groupings and to reduce the control of the National Office, may indeed be a move in the right direction. However, the new risk lies in creating a silo effect with *headspace* centres being run individually, without the right encouragement of interconnections between *headspace* centres. Complexity science teaches that flexible interaction between stakeholders is a perennially desirable feature of systems.

A final pointer to better care integration in mental health, inspired by CAS theory, could be to not see something like the *headspace* initiative as the ultimate end, *the* solution to integrated care. Rather such an initiative should be regarded as an iterative process, a service always in ongoing development, which will require refinement and adaptation both to local contexts and dynamically over time. Feedback, the propensity for a CASs outputs to then become inputs to the behavior of the system can be harnessed in this regard, by communicating back to those working at *headspace*, at the local level, not only what goes wrong but right, too [23]. Ensuring service-users have a prime position in this feedback loop, that the quality of their experience within this system is the major source of both output and input, will further enshrine their attractor status, and help guide longitudinal and flexible service improvements.

## Conclusion

CAS principles are being applied to health care elsewhere [6, 11, 13]. The theory behind complexity helps characterise what systems have in common (e.g., individual agents, self-organization, emergent behaviours, dynamic changes over time, and localised rather than imposed solutions). These features can be leveraged to support improved care [13]. Complexity science doesn’t make the task of enabling services any easier; the complexity of the systems cannot be wished away or tamed. While it is by no means a magic bullet, it can, however, help clarify the magnitude of the task of joining up services, and provide pointers to the efforts needed to create more connected care for clients with mental health conditions.

What it does mean is that there is a critical decision needed to leverage the benefits of the CAS features: to be much more patient-focused; allow care to be built on partnerships; for the system to be run more bottom-up than top-down, prescribing what should happen from the upper echelons of the system. This has traditionally been hard for publicly funded systems which have the constant urge to “manage” services from the system’s apex, and invoke hierarchical models of care. To make the shift in thinking that is called for, the theory of the CAS points us in the direction of what we need to do.
